# Systematic Comparison of the Effect of Four Clinical-Grade Platelet Rich Hemoderivatives on Osteoblast Behaviour

**DOI:** 10.3390/ijms20246243

**Published:** 2019-12-11

**Authors:** Tulio Fernández-Medina, Cedryck Vaquette, Sašo Ivanovski

**Affiliations:** 1 School of Dentistry, The University of Queensland, Herston, QLD 4006, Australia; c.vaquette@uq.edu.au; 2 School of Dentistry and Oral Health, Griffith University, Southport, QLD 4215, Australia

**Keywords:** platelet rich plasma, platelet rich fibrin, bone formation, growth factors, standardisation

## Abstract

Hemoderivatives have utilized in an empirical manner, driven by clinical considerations, leading to the development of a plethora of manufacturing protocols. The purpose of this study was to investigate the composition and bioactivity of four common clinical-grade hemoderivates prepared using standardised methods. Four different hemoderivatives were obtained from sheep blood and divided into two groups: A-PRF/i-PRF (fresh) and P-PRP/L-PRP (anticoagulated). Thrombus (CLOT) was used as a control. Thrombocyte quantification, growth factor composition (IGF-I, VEGF, PDGF-BB, BMP-2), cell viability, migration and mineralization assay were evaluated. Platelet recovery was superior for L-PRP followed by P-PRP. A significant cumulative release of IGF-I and PDGF-BB was noted for A-PRF and L-PRP groups at early time points. Similar release profiles of BMP-2 and VEGF were noted in all protocols. Cell viability and migration assay have demonstrated a detrimental effect when the concentration was ≥60%. Moreover, at Day 21, i-PRF have demonstrated superior mineralisation properties when compared to all groups. A negative impact of A-PRF was demonstrated at high concentrations. Despite its low content in growth factors, i-PRF was the best performing blood product for inducing osteoblast mineralisation, and therefore could be the candidate of choice for utilisation in bone tissue engineering applications.

## 1. Introduction

Hemoderivative preparations are obtained via blood plasma fractionation through centrifugation. Their utilisation for tissue healing enhancement in a wide range of clinical applications have been reported in the literature since the early 1950s [1]. The action of these preparations relies on the secretion of a cocktail of proteins from platelet-α-granules. As autologous formulations, prepared via relatively simple chairside fabrication, these blood products have recently generated considerable interest in clinical regenerative medicine [2,3,4,5]. Growth factors, such as insulin-like growth factor-1 (IGF-I), platelet-derived growth factor (PDGF), vascular endothelial growth factor (VEGF), fibroblast growth factor (FGF), epidermal growth factor (EGF), platelet-derived epidermal growth factor (PDEGF) and fibrin matrix proteins, are found in these preparations at a higher concentration than in blood, and have been proposed to directly contribute to accelerating tissue regeneration [5,6,7]. Hemoderivative preparations have also been frequently used as a surgical adjuvant to improve healing and promote tissue regeneration in the treatment of intrabony defects [8,9,10], alveolar ridge augmentation [11], post-extraction socket preservation [12], treatment of membrane perforation [13] and maxillary sinus augmentation [14,15].

Several different hemoderivative products can be distinguished, as classified by Dohan et al. [16]. This classification is based on a product’s cellular content (mainly leukocytes) and fibrin architecture, resulting in four main families: (1) Pure platelet-rich plasma (P-PRP)—or leukocyte-poor platelet rich plasma (LP-PRP); (2) Leukocyte-and platelet-rich plasma (L-PRP); (3) Pure PRF (P-PRF)—or leukocyte-poor platelet-rich fibrin; and (4) Leukocyte- and platelet-rich fibrin (L-PRF). With the exception of L-PRF, these preparations propose the inclusion of anticoagulant and activator agents (i.e., CaCl_2_) to obtain the platelet concentrate. However, the inclusion of the anticoagulant agents, as well as calcium chloride-induced fibrin polymerization and hard-centrifugation (≥210 *g),* which affects the quantity and quality of platelet recovery and growth factor release [17,18], may significantly influence the healing behavior compared to natural fibrin clotting. Platelet lysates have also been proposed as supplements for cell proliferation and expansion [19] but their clinical applicability is still controversial due to the risk of contamination during manufacturing that occurs away from the chairside [20,21].

In a more recent development, another hemoderivative family has been reported in a series of papers, describing the so-called Advanced Platelet Rich Fibrin (A-PRF) [22,23,24,25]. This new generation of platelet concentrate, which is prepared without anticoagulants and involves a single soft-centrifugation step method leading to the rapid formation of a fibrin network, has demonstrated a promising wound-healing capacity [22,24]. A-PRF is typically processed in the form of a membrane and has been claimed to contain equivalent amount of platelets and almost 50 per cent of the leukocytes present in the initial blood harvest, embedded within a strongly polymerized fibrin matrix [26]. Subsequently, an injectable version was developed by Mourão et al. [27], proposing a method to produce a liquid platelet rich fibrin (i-PRF) which could be injected before gelation. Similarly to A-PRF, the preparation method involves a short soft centrifugation of fresh blood without the addition of an anticoagulant, thus enabling the harvesting of an orange coloured plasma on the surface of the collection tube [28]. These recent advances in the fabrication of hemoderivatives have resulted in the introduction of the “Blood Concentrates” concept, as opposed to “Platelet Concentrates” (such as those of the PRP family), whereby the soft centrifugation enables the inclusion of additional cellular components such as circulating stromal cells and white blood cells entrapped in the liquid i-PRF [29].

The development of various hemoderivatives has been generally based on empirical methods, whereby clinical handling and ease of implantation were the main selection criteria. As a result, and in addition to inherent patient-to-patient variations, the field is marred by a lack of manufacturing standardisation, which is likely to be responsible for the contradictory results observed in vivo. These regenerative discrepancies originate from small, but consequentially significant, technical modifications such as variation in *g*-force, time of centrifugation, centrifuge characteristics and selective inclusion-exclusion of the buffy coat layer [7]. This lack of standardisation is responsible for the great variations in the biological composition within a given type of hemoderivative, and hence hinders meaningful and scientific comparison across the various types of blood products.

Therefore, the objective of the present study was to manufacture, in a standardised and highly controlled manner, four clinically utilized hemoderivatives, using a commercially available clinical grade centrifuge. These hemoderivatives were systematically characterized to determine their biological composition and assess their effect on osteoblast function.

## 2. Results

### 2.1. Thrombocyte Quantification (Table 1)

Total thrombocyte numbers were determined for whole blood and the various hemo-preparations. Similar platelet recovery was observed for total blood (362 ± 22 × 10^3^) and natural clot (326 ± 32 × 10^3^) (Table 1). Comparable results were observed for A-PRF (307 ± 14 × 10^3^) and i-PRF (336 ± 18 × 10^3^). P-PRP platelet recovery was 3-fold higher (934 ± 32 × 10^3^) compared to total blood and the highest numbers were quantified for L-PRP (1.82 ± 17 × 10^6^).

### 2.2. Release of Growth Factors from Different Hemoderivative Preparations (Figure 1)

The release profiles of IGF-I, PDGF-BB, VEGF and BMP-2 from the different platelet rich protocols were investigated at various time points (1, 3, 7, and 14 days).

**Figure 1:**
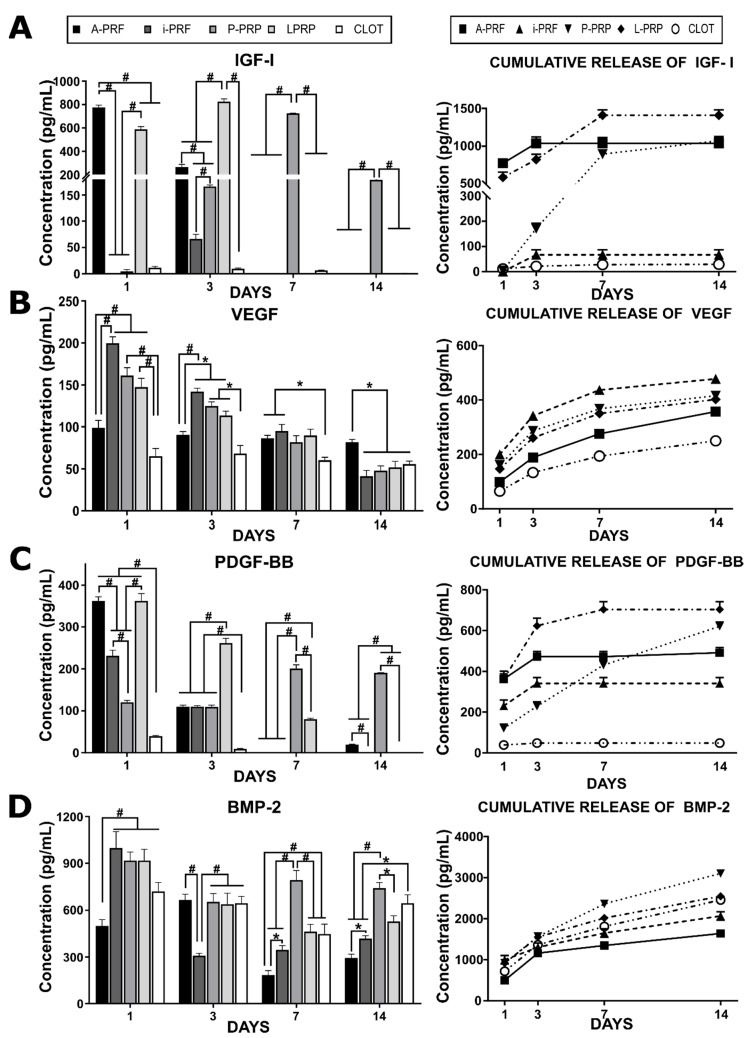
Growth factors release profiles from the five different hemoderivatives over 14 days. (**A**)—Insulin Growth Factor I (IGF-I); (**B**)—Vascular Endothelial Growth Factor (VEGF); (**C**)—Platelet-derived growth factor BB (PDGF-BB); (**D**)—Bone Morphogenetic Protein 2 (BMP-2). The left hand side graphs show release at individual time points (1, 3, 7, 14 days), while the right hand side shows cumulative release profiles over the 14 day duration of the experiment. Stars indicate statistically significant differences (* *p* < 0.05; # *p* < 0.001).

IGF-I was rapidly released from A-PRF within three days post incubation (Figure 1). L-PRP demonstrated a more sustained release profile, with a third of the total IGF-I released by 24 h, followed by a gradual release until seven days post-incubation. Interestingly, a delayed release was observed for P-PRP, which displayed no release of IGF-I during the initial 24 h, followed by a linear release until seven days. Interestingly, i-PRF and the blood clot did not demonstrate any significant release of IGF-1 at any of the time points considered.

A different trend was observed for VEGF, whereby all hemoderivatives displayed a sustained release over the entire 14 days of the experiment. The total amount of VEGF released by the hemoderivatives did not differ greatly (around 400 pg/mL for each), however, release from the blood clot was significantly lower than in all of the other groups (Figure 1B).

PDGF was rapidly released from the “fresh” hemoderivatives A-PRF and i-PRF, with the majority of this growth factor released after three days of in vitro incubation. A similar trend was observed for L-PRP, although the total amount of PDGF released was significantly higher, reaching over 600 pg/mL. Interestingly, P-PRP displayed a linear release of PDGF for the entire 14 days, also reaching total levels around 600 pg/mL.

The release profile of BMP-2 appeared very homogenous from the various hemoderivatives, with a sustained release over the entire length of the experiment. However, the total release of this growth factor differed, with the highest amount released by P-PRP, intermediate amounts for the blood clot and L-PRP, and the lowest release levels by i-PRF and A-PRF.

Aside from the BMP-2 release profile, where A-PRF and i-PRF underperformed, all of the hemoderivatives demonstrated statistically significant superiority when compared to a natural clot for the total release of the most important growth factors for wound healing, angiogenesis and bone formation.

### 2.3. Cell Viability (Figure 2)

**Figure 2:**
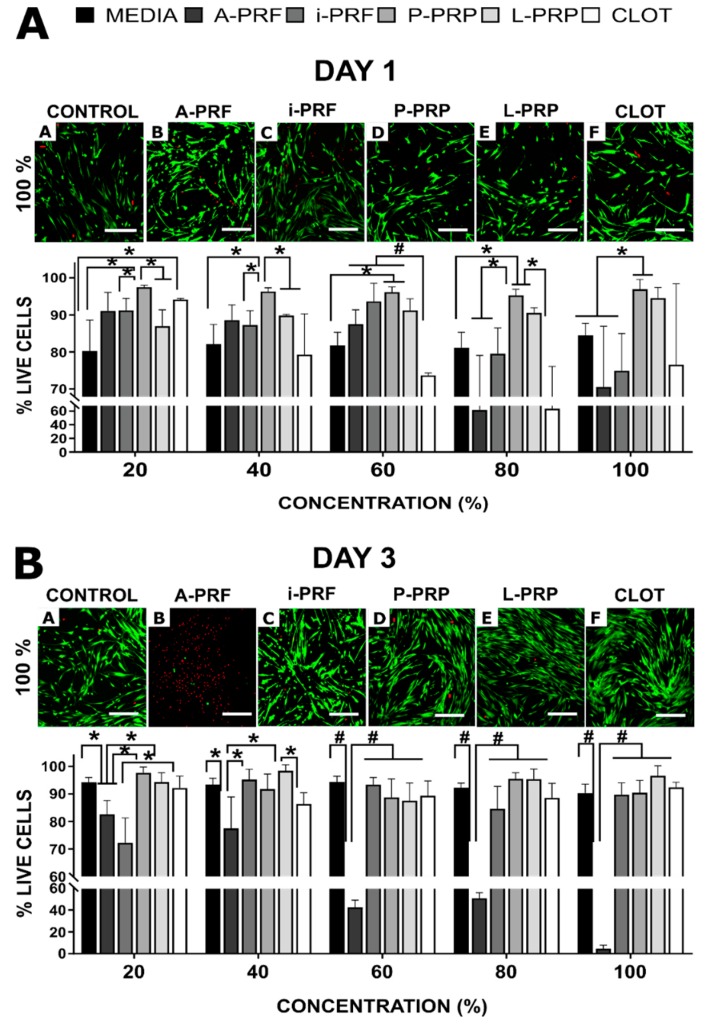
Osteoblast viability. Confocal images of live-dead staining and percentage cell viability after culture in various concentrations of hemoderivative exudate after (**A**) 1 day and (**B**) 3 days. Scale bar at 100 μm; green shows living cells and red shows dead cells. Stars indicate statistically significant differences (* *p* < 0.05; # *p* < 0.001).

Cell viability assessment at Days 1 and 3 revealed that A-PRF and CLOT were cytotoxic at high concentrations (Figure 2). At 20% concentrations, A-PRF showed similar behaviour to the other groups (viability = 91 ± 5), but once the concentration increased beyond 80%, cell viability significantly decreased, even at 24 h (for e.g., 100% A-PRF = 71 ± 16% viability). A similar trend was observed for the CLOT, presenting a decreased viability for high concentrations (from 60% (74 ± 1) to 100% (76 ± 22). The A-PRF cytotoxicity was particularly evident at three days for concentrations above 60%, ranging from 88 ± 4 (60%) to 5 ± 3 (100%). The anticoagulated products (L-PRP and P-PRP) were superior (*p* < 0.05) at a 60% concentration or higher, when compared to the control blood clot at day 1. Moreover, i-PRF induced a reduction in cell viability at Day 1 when compared with L-PRP and P-PRP (*p* < 0.05). However, by Day 3 and at concentrations greater than 20%, i-PRF was comparable with the rest of the hemoderivates, apart from the aforementioned A-PRF group (*p* < 0.001).

### 2.4. Cell Metabolic Activity 

**Figure 3:**
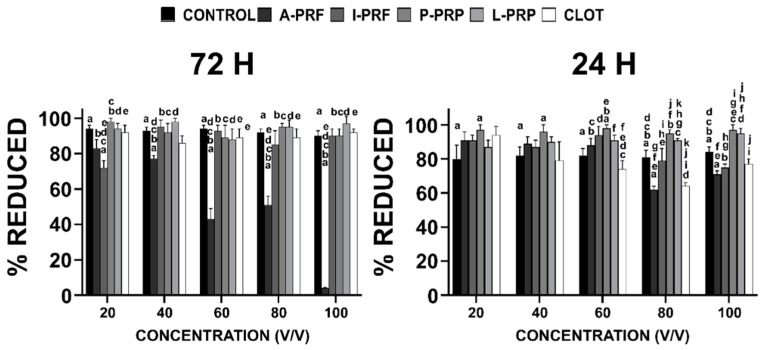
Alamar Blue^®^ assay. Percentage of reduction in osteoblast metabolic activity after culture in various concentrations of hemoderivative exudate at 24 and 72 h. The letters represent the statistical difference between groups (*p* < 0.05).

Cell metabolic activity at 24 and 72 h showed that A-PRF was cytotoxic at high concentrations (Figure 3). At a 20% concentration, A-PRF showed similar behaviour to the other groups (% Reduction = 85 ± 5), but negatively affected metabolic activity for concentrations higher than 80% (cell metabolic activity significantly decreased at 24 h, with a percentage of reduction of 71% ± 16% for APRF 100%). A similar trend was observed for the CLOT group, presenting a decreased metabolic activity at high concentrations (% reduction at 80% was 64% ± 12%, and at 100% it was 77% ± 22%). The A-PRF cytotoxicity was particularly evident at three days for concentrations above 60%, ranging from 43% ± 6% (60%) to 4% ± 3% (100%). The anticoagulated products (L-PRP and P-PRP) promoted higher cell metabolic activity when compared to the control group for concentrations above 80% at Day 1.

Notably, i-PRF initially (at Day 1) induced a reduction in metabolic activity for concentrations above 80% when compared with L-PRP and P-PRP. However, by Day 3 and for concentrations greater than 40%, i-PRF was comparable with the rest of the hemoderivates.

### 2.5. Cell Migration 

**Figure 4:**
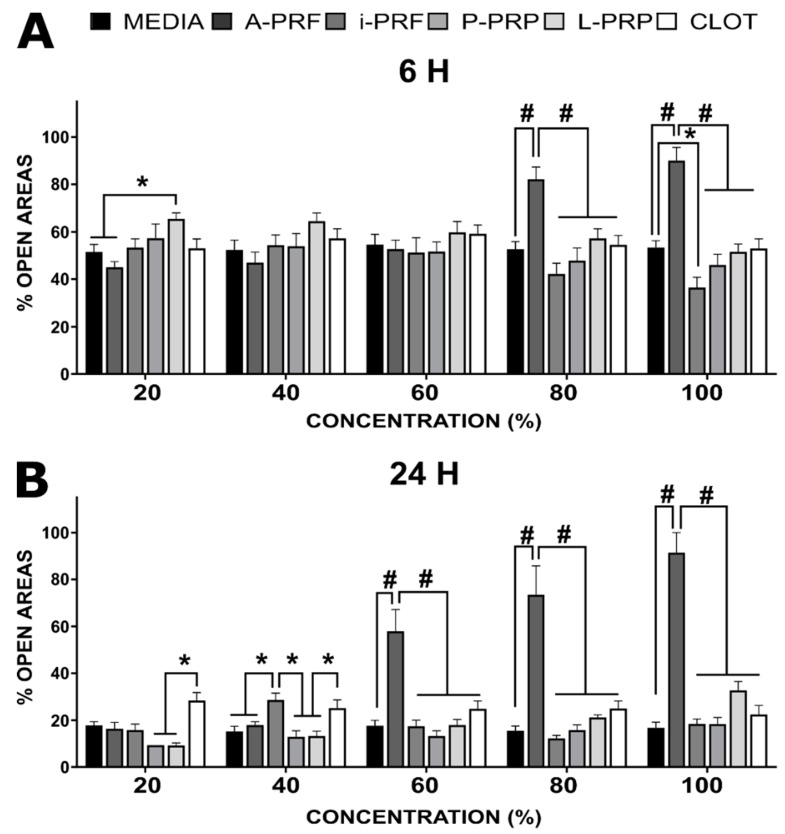
Percentage of open areas of the scratch-osteoblast migration assay in response to the various concentrations of the hemoderivatives. (**A**) 6 h; (**B**) 24 h post-incubation. The stars (*) indicate statistical significance (* *p* < 0.05/# *p* < 0.001).

Consistent with the cell viability results, the migration of osteoblasts (% of open areas) was impeded by higher concentrations of A-PRF, i.e., ≥80% at 6 and ≥60% at 24 h post incubation (Figure 4). However, early time points have demonstrated superior behaviour for media (52 ± 3) and A-PRF (45 ± 2), compared to P-PRP (57 ± 6) at 20% dilution. Similarly, i-PRF (36 ± 4) resulted in superior osteoblast migration compared to media (51 ± 3) at 100%. At a later timepoint, P-PRP (9 ± 1) and L-PRP (9 ± 1) have demonstrated superior performance compared to CLOT (28 ± 3) at 20% dilution. This trend was also observed at 40%, in conjunction with the inferior behaviour of i-PRF (29 ± 3) compared to all groups except CLOT (25 ± 3). From this point, A-PRF presented a detrimental effect on cell migration compared to all groups when concentrations were 60% or higher (*p* < 0.0001). There were no other differences between the groups at higher concentrations after 24 h.

### 2.6. Mineralization Assay 

**Figure 5:**
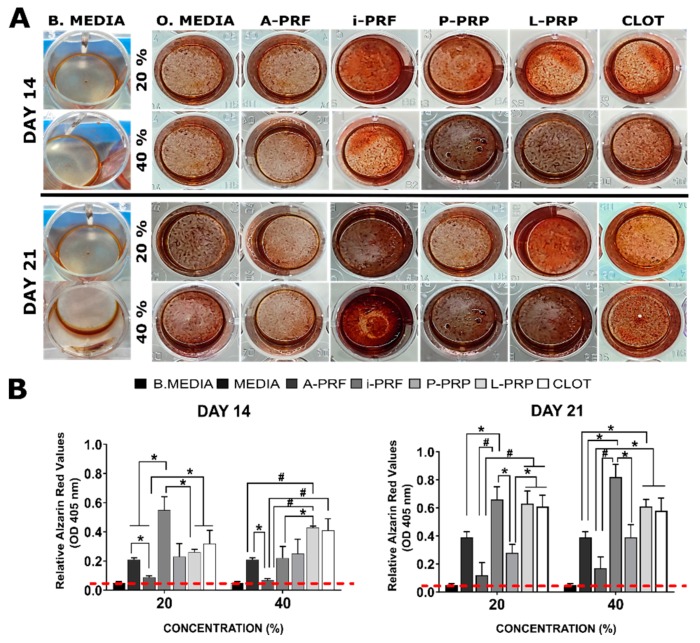
Alizarin Red staining of osteoblast in response to various concentrations of the hemoderivative exudates, basal (negative control) and osteogenic (positive control) media groups. (**A**) optical observation of the staining at 14 and 21 days for the concentration of 20% and 40%. (**B**) Quantification of the mineralisation at 14 and 21 days. The stars indicate statistical significance when compared to control (* *p* < 0.05/# *p* < 0.001). Dotted red line indicates the basal media relative Alizarin value (OD 405 nm).

The mineralization (Alizarin Red/OD 405 nm) assay was conducted with hemoderivative concentrations of 20% and 40% (*v*/*v*) (Figure 5). Basal media without osteogenic factors (0.05 ± 0.01) was applied as negative control. At Day 14 in 20% dilution i-PRF (0.5 ± 0.1) demonstrated superior behaviour compared to all groups except for CLOT (0.3 ± 0.1) and P-PRP (0.2 ± 0.02). Additionally, A-PRF (0.1 ± 0.07) significantly inhibited mineralization and demonstrated an inferior mineralisation capacity when compared to all groups (* *p* < 0.05) except for P-PRP. This trend was maintained at 40% for A-PRF, showing reduced behavior, compared to osteogenic media (* *p* < 0.05), L-PRP and CLOT (# *p* < 0.001). Similarly, L-PRP (0.4 ± 0.01) have shown superiority compared to i-PRF (0.2 ± 0.08) and osteogenic media (0.2 ± 0.01). At Day 21 in 20%, i-PRF (0.6 ± 0.1), L-PRP (0.6 ± 0.1) and CLOT (0.6 ± 0.1) have demonstrated a significant superior capacity to induce mineralisation compared to P-PRP (0.3 ± 0.1) and A-PRF (0.2 ± 0.1). On the contrary, osteogenic media (0.4 ± 0.1) have demonstrated inferior behaviour only when compared to i-PRF. Moreover, at higher concentrations, (40%) i-PRF displayed increased mineralisation (0.8 ± 0.1) compared to P-PRP (0.4 ± 0.1), A-PRF (0.2 ± 0.1) and osteogenic media (0.4 ± 0.1). Similar difference (* *p* < 0.05) was observed for L-PRP compared to A-PRF and osteogenic media. CLOT have shown differences only when compared to A-PRF (* *p* < 0.05).

### 2.7. Growth Factors Concentration Vs. Cell Viability Correlation 

**Figure 6:**
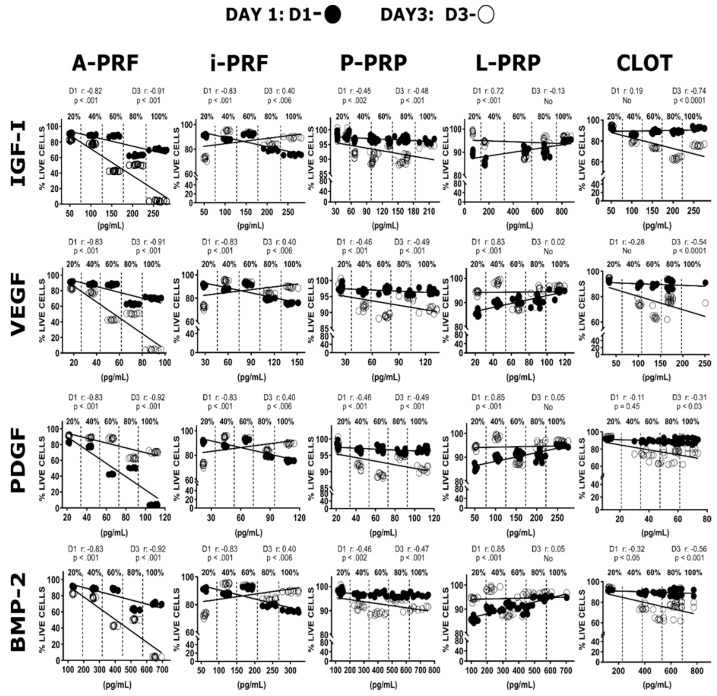
Correlations of the different growth factor content on A-PRF, i-PRF, P-PRP, L-PRP and CLOT dilutions (20%, 40%, 60%, 80% and 100%) and osteoblast viability at one and three days. This reveals the detrimental effect of A-PRF group when concentrations were higher than 40%.

Multiple correlations were performed to investigate the effect of autologous growth factors concentration (20%, 40%, 60%, 80% and 100%) within the different hemoderivates on osteoblast viability (Figure 6). Pearson correlation coefficient has a value between +1 and −1: the closer the value is to 1 or −1, the stronger the correlation, which may imply a causal link (positive or negative) between concentration and cell viability [30]. Generally, the patterns of correlation were characteristic for a given hemoderivative, with the type of growth factor or their specific concentration only having a minor effect. There was a strong negative correlation between growth factor concentrations and cell viability for A-PRF at both one (*r* = −0.8) and three (*r* = −0.9) days. A negative correlation was also observed at Day 1 for i-PRF (*r* = −0.8), which subsequently increased but still remained a weak positive correlation at Day 3 (*r* = 0.4). On the contrary, there was a strong positive linear correlation for the L-PRP group at Day 1 (*r* = 0.8) but virtually no correlation at Day 3 (*r* = 0.1). The P-PRP group showed only a weak negative correlation at Days 1 and 3 (*r* = −0.5). Thrombus (CLOT) have demonstrated almost no correlation for the different growth factors after one and three days (ranging from *r* = −0.7 to *r* = 0.2). 

### 2.8. Correlation between BMP-2 Levels and Mineralization 

**Figure 7:**
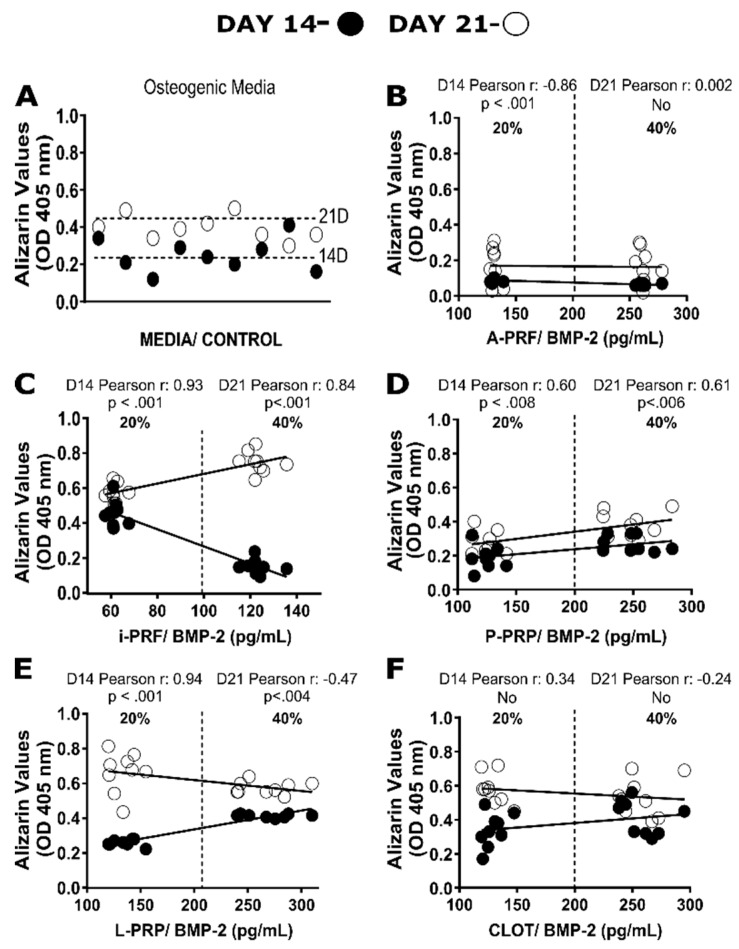
Scatter plot correlations between the Alizarin Red quantification on osteoblast and bone morphogenetic protein-2 (BMP-2) content for control (**A**), A-PRF (**B**), i-PRF (**C**), P-PRP (**D**), L-PRP (**E**) and clot (**F**) after 14 and 21 days.

Here again, multiple correlations were performed to evaluate the impact of autologous BMP-2 levels within the different hemoderivates (20% and 40% dilution) at 14 and 21 days (Figure 6) and their capacity to induce mineralization (Figure 7). The A-PRF group demonstrated a strong negative correlation at Day 14 (*r* = −0.9) and almost no correlation at Day 21 (*r* = 0.1). The i-PRF group showed a strong positive correlation at 14 (*r* = 0.9) and 21 (*r* = 0.8) days. Moreover, P-PRP was associated with almost no correlation (*r* = 0.6) at Days 14 and 21. Additionally, the L-PRP group exhibited a high positive linear correlation (*r* = 0.9) at Day 14, but had almost no correlation at 21 days (*r* = −0.5). Although the natural clot has exhibited comparable alizarin red values (0.6 ± 0.1) to i-PRF and L-PRP at 14 days (20% dilution) it showed almost no correlation at 14 (*r* = 0.4) and 21 *(r* = −0.3) days.

## 3. Discussion

Platelet rich preparations have been extensively reported in the literature within the past two decades to promote healing in a variety of clinical applications [12,31,32]. A systematic scientific comparison of the different types of hemoderivatives is an arduous task, due to the intrinsic biological variations in autologous products but also due to a lack of standardisation of the various protocols. The results reported in the literature are contradictory and the subsequent regeneration efficacy of these blood products remains debatable, especially in the context of bone formation [14,15]. Indeed, platelet rich fibrin products are osteoinductive (the capacity to induce ectopic bone formation) [33], but the bone regeneration properties of these hemoderivatives in combination with particulate bone substitutes have shown divergent outcomes [10,11,15,34]. This could be attributed to variations in the implantation sites or is also likely to be due to differences in the blood product manufacturing methods. Therefore, this lack of consistency across the protocols for the preparation does not allow for direct comparison between different studies and/or the various hemoderivatives. The development of these products has historically been performed chairside, and hence clinical considerations, such as handling, mechanical properties, manufacturing time, etc., have taken precedence over the systematic and comprehensive evaluation of biological performance. Hence, more insight is necessary for achieving a better understanding of the performance of these blood products.

Another alternative recently proposed is the incorporation of human platelet lysate (HPL) as clinical-grade supplement for cell expansion and cell therapy [19,20]. The biological effect relies on the high concentration of growth factors released from platelets by sequential freeze/thaw cycles. Despite the promising healing HPL properties, the main drawback is the potential risk of transmitting highly pathogenic lipid-enveloped viruses such as hepatitis B virus (HBV), hepatitis C virus (HCV) and human immunodeficiency virus (HIV) [35]. The need for processing lysate outside of the chair-side setting dramatically increases the risk of sample contamination and subsequent utilisation in translational regenerative therapies [21]. These drawbacks can be mitigated by using chair-side hemoderivates such as P-PRP, L-PRP or i-PRF, which are processed chairside in closed systems, thereby reducing the possibility of cross-contamination. Recent attempts to assess the performance of HPL compared to traditional PRP [36] have demonstrated that high concentrations of growth factors, such as TGF-β1, VEGF, PDGF-AB, in platelet lysate, did not result in increased cell viability or extracellular matrix protein gene expression. It was hypothesised that some of the growth factors involved in cell proliferation could be altered or degraded during the freezing step at −80 °C, resulting in a reduced impact on cellular behaviour. The clinical application of hemoderivates that can reliably deliver growth factors while reducing the possibility of sample contamination is a major requirement for the incorporation of blood products in tissue-engineering strategies.

Our findings confirmed that platelet recovery is strongly correlated to the parameters applied during the manufacturing of the various hemoderivatives, displaying that greater amounts of thrombocytes were generated from protocols which incorporated a prolonged centrifugation time and higher centrifugation force [37]. However, the derived growth factor compositions in the resulting blood products did not necessarily correlate with total platelet levels. For example, while L-PRP displayed a 1000-fold increase in platelet recovery, its growth factor content was not substantially higher compared to the other groups (produced with lower *g*). This suggests that the behaviour of these blood products is more complex and extends beyond the well-described platelet role [7,25]. It appears that the differential fibrin structural network, plasma protein content, hormones and electrolytes, as well as variations in cell populations resulting from the inclusion/exclusion of the buffy coat layer content, could be responsible for this heterogeneous biochemical composition and release profile. For example, the release of the growth factor can be strongly influenced by the structure of the fibrin network and its biochemical interaction, indeed, the fibrin network has been hypothesised to act as a reservoir and is likely responsible for the controlled release of growth factors and cytokines [25,38].

In addition to the kinetics of release, the global composition of the hemoderivatives can also play a direct role in the subsequent cellular response, as growth factors can induce both anabolic and catabolic effects in cell behaviour [39]. Similarly to a previous study [40], the dosage of biochemical cues demonstrated a strong influence on cellular survival, function, and higher concentrations of hemoderivative exudates, especially for A-PRF, i-PRF and CLOT at 100% after 24 h post incubation, which are associated with detrimental effects. Nevertheless, this effect disappeared for all groups except A-PRF at Day 3. Furthermore, our results suggest that any toxicity is reduced when the exudates are utilised at lower concentrations, indicating a dose-dependent behaviour rarely highlighted in the literature. Most of the studies investigating the effect of hemoderivatives on cell behaviour have used a 20% concentration of the exudates for technical reasons (availability and quantity of blood, etc.) or to allow for comparison to the established literature [6,7,41,42]. Ultimately, an ideal in vitro or in vivo concentration has yet to be defined, and the result of this study was able to provide a unique insight into the effect that various concentrations common hemoderivative preparations have on cell function.

The findings challenge some of the current clinical practices utilising A-PRF, as it is customary to mix the A-PRF exudate (produced after the manufacturing of the A-PRF membrane) with particulate bone substitutes [8,43,44]. Our results clearly demonstrated cytotoxicity, as well as an impediment of cell migration and mineralization, for higher concentrations of this blood product. It is therefore possible that various cellular (i.e., monocytes, lymphocytes, MSCs) and molecular components, aside from thrombocytes [29], that are entrapped in the fibrin A-PRF network may produce these adverse biological side effects, which are not fully described or understood. The type of fibrin mesh resulting from the A-PRF preparation could explain this adverse effect. After performing the centrifugation cycle (208 g × 8 min), the blood tubes are rested vertically for 5 min. This allows for the natural spontaneous clotting of the A-PRF, which is reflected in its composition: the centre of the clot is composed of a stable and dense fibrin network surrounded by a soft and partially polymerized fibrin structure. The outer fibrin layer may not be completely cross-linked, thus producing a weak fibrin mesh structure that is not able to contain all of the biochemical cues, proteins and growth factors, which are then released in a burst release profile into the media. This hyper-concentrated growth factors media may result in a cytotoxic environment under in vitro conditions. This hypothesis is in agreement with previous reports that highlight the role of the fibrin network in the release kinetics of growth factors [3,45,46].

There was a remarkable mineralisation capacity for i-PRF at both the early and late time points, displaying approximately 2-fold increase when compared to the control group. Similar results were reported previously [41], showing the superior mineralisation potential of i-PRF over L-PRP. The in-vitro results could explain the positive bone regeneration outcomes when i-PRF is utilised as a supplement [8,11]. However, this finding is not entirely consistent with the biochemical composition measured in the i-PRF exudate (after three days of incubation). Indeed, i-PRF had some of the lowest growth factor concentrations and this was particularly notable for BMP-2, a known osteogenic cue. These findings suggests that i-PRF may produce a protein cocktail, whereby the different biochemical cues, although in smaller amounts, may work in a synergetic manner, leading to a positive biological impact for tissue regeneration [39]. This hypothesis needs to be evaluated in an in vivo model.

The correlation analysis confirmed the negative linear relationship between higher A-PRF concentrations (>40%) and cell viability at one (*r* = −0.8) and three (*r* = −0.9) days. Interestingly, the same growth factors resulted in a superior cellular performance at higher concentrations in other hemoderivative groups (i.e., P-PRP and L-PRP), suggesting that the final biological response is not solely related to the amount of secreted grown factors, and perhaps the combination of different biological cues, matrix components and even cellular content could be responsible for a given cellular response. Our finding contrasts with a previous report [47] that demonstrated the superiority of A-PRF (approximately 100% viability) compared to control after three days of culture. Although these authors used 20% A-PRF dilution, the standard DMEM media was supplemented with 15% FBS, raising questions about whether such a biological impact could be attributed to the hemoderivate by itself, rather than the combination of different serum concentrations. Nevertheless, it was also demonstrated that highest proliferation rate of the MGC3 cell line is correlated with the presence of lower PRP concentrations, ranging between 10% and 25%, suggesting that an increase in hemoderivate concentration has a detrimental effect on proliferation rate [48]. Wang et al. [41] also confirmed this successful hemoderivate low concentration strategy, comparing the cell viability of P-PRP and i-PRF at 20% dilution, showing 200% and 280% higher capacity than the control, respectively. This behaviour was also accompanied by approximately 2-fold mineralisation capacities in favour of i-PRF when compared to the control.

Moreover, growth factor concentrations showed considerable differences between different hemoderivates at the same dilution. For instance, secreted IGF-I levels were ≈50 pg/mL in 20% dilution for the A-PRF and i-PRF groups, while P-PRP (≈30 pg/mL) and L-PRP (≈10 pg/mL) released different amounts at the same dilution. The presence of these variations, even with the high level of standardisation in this study, highlights the challenge of predicting the biological behaviour of hemoderivates. Indeed, while a strong positive linear correlation of i-PRF BMP-2 concentrations enhanced mineralisation at both 1(*r* = 0.9) and 3 (*r* = 0.8) days, it should be noted that this effect was produced by a lower BMP-2 concentration (≈60 pg/mL) compared to other groups (≈125 pg/mL). Recently, Anitua and co-workers [38] introduced the concept of autologous platelet-and plasma-derived protein fibrin scaffolds (ABDPS) and highlighted the importance of this hemoderivate in minimising the growth factor dose, with the capacity to produce a similar biological effect compared to recombinant, commercially available cytokines at higher concentrations. This dose–effect mismatch was observed by comparing clinical and pre-clinical studies, showing that doses of autologous GFs are capable of inducing positive effects in human skin ulcer healing (6 mm diameter) using autologous VEGF (8 ng) and PDGF (640 ng) within ABDPS [49]. Surprisingly, similar mice skin ulcers (5.5 cm diameter) have required superior concentrations of recombinant VEGF (20 μg) or PDGF-BB (10 μg) to effectively heal [50]. This behaviour highlights the importance of the dose–effect relationship in clinical applications of the aforementioned autologous blood concentrates.

Aside from clinical considerations, the nature of the protocol used for chairside preparation, and variations in blood composition across patients, it appears that poorly characterised and understood biochemical cues not directly related to platelet or growth factor concentrations can significantly impact the cell and tissue response to hemoderivatives. The autologous nature of hemoderivatives implies a lack of immunogenic or other adverse biological effects and, as such, provides the clinical rationale for their use. However, there is evidence that variations in biochemical composition, matrix structure and configuration, as well as a variety of entrapped cell populations, could evoke an unknown and potentially adverse interaction with the recipient’s tissue. Therefore, further studies are required to characterize the tissue immune–inflammatory response to these “healing enriched environments”, in addition to understanding how variations in composition can be modulated to produce a desirable response under in-vivo conditions.

## 4. Materials and Methods 

All experiments were conducted in accordance with the guidelines described by *the Australian Code of Practice for the Care and Use of Animals for Scientific Purposes* and received approval from the Queensland University of Technology Animal Ethics Committee (approval number: 1500001242; approval date: 15/02/2017) and ratified by The University of Queensland (approval number: QUT 1500001242; approval date: 30/05/2018).

### 4.1. Blood Products (A-PRF, i-PRF, P-PRP, L-PRP and Natural Clot) (Figure 8)

Ovine blood samples from Merino sheep (3–4 years old) were collected from three different animals and freshly prepared in triplicate immediately after blood was drawn, prior to coagulation. All blood products were manufactured using a commercially available clinical centrifuge (Duo Centrifuge, Fixed angle rotor/radius 110 mm, Biomedent Australia).

### 4.2. Fresh Processing Protocols (A-PRF, i-PRF and Natural Clot)

**Figure 8:**
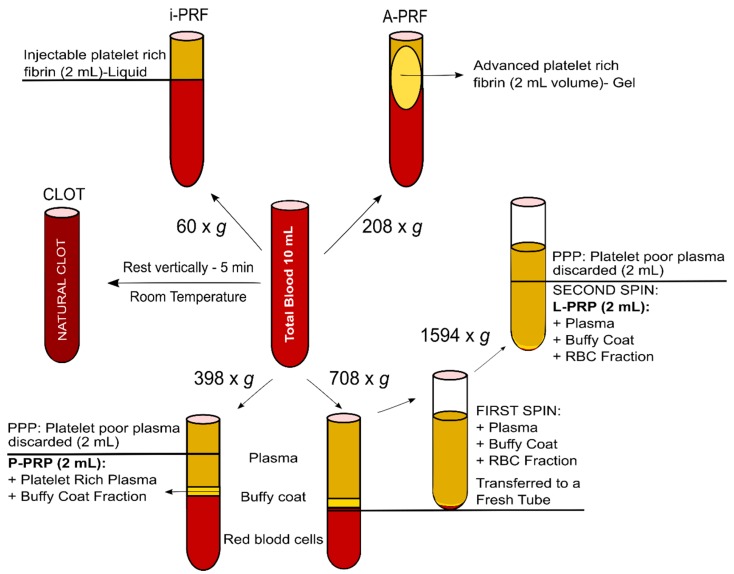
Protocols for the preparation of the various hemoderivative products. A commercially available clinical centrifuge was utilized to process the products under standardized conditions, including volume of blood, tubes and temperature.

A-PRF and i-PRF were prepared according to established protocols, utilised routinely in the clinical setting [22,29] and summarized in Figure 8. In order to maintain a high degree of standardisation, 10 mL of blood was utilised for the preparation of each specimen.

#### 4.2.1. A-PRF Preparation

For A-PRF, fresh blood was collected in a 10 mL vacuum tube (Vacuum sterile plain tubes, A-PRF™ Biomedent. Cove West NSW, Australia) and immediately centrifuged at 1300 rpm (208 g) for 8 min, followed by a 5 min resting step. Thereafter, the clot was extracted and the red blood cell fraction was carefully removed using surgical scissors. The resulting A-PRF (2 mL volume) was placed in a 6-well-plate with 5 mL of alpha-minimum essential media (α-MEM, Thermo Fisher Scientific Inc. Newstead QLD, Australia) supplemented with 1% reduced Fetal Bovine Serum (FBS; Gibco, Thermo Fisher Scientific Inc. Newstead QLD, Australia), 1% antibiotics (100 U/mL penicillin G, 100 μg/mL streptomycin, Thermo Fisher Scientific Inc. Newstead QLD, Australia) and 1% of non-essential aminoacids (Gibco^®^ Thermo Fisher Scientific Inc. Newstead QLD, Australia), which is referred to as serum-reduced media for the remainder of this study.

#### 4.2.2. i-PRF Preparation

For i-PRF, 10 mL of blood was also collected in a vacuum tube (Vacuum tube i-PRF^TM^ Choukroun, Biomedent. Cove West NSW, Australia) and centrifuged at 700 rpm (60 g) for 3 min. Following centrifugation, the orange supernatant (2 mL), representing the liquid i-PRF, was aspirated using a syringe fitted with a 21G needle and placed in a 6-well-plate until natural gelation occurred. Thereafter, 5 mL per well of serum-reduced media was added into the gelled product.

#### 4.2.3. Blot Clot Preparation

Natural blood clots were obtained from blood collected in 10 mL vacuum tubes and left to rest for 5 min in a vertical position for clotting to occur. Thereafter, a 2 mL volume of total blood clot was carefully removed using surgical scissors and transferred to a 6-well-plate with 5 mL of serum-reduced media.

### 4.3. Anticoagulated Blood Protocols (L-PRP and P-PRP)

Anticoagulated preparations were performed, following protocols described previously [34,37]. Briefly, 10 mL of blood was collected using sodium-citrated tubes (Livingstone International PTY LTD. Rosebery NSW, Australia). The collected blood was manually and gently stirred to prevent coagulation and ensure homogeneity of the sample prior to further processing.

#### 4.3.1. L-PRP Preparation

The preparation of the L-PRP samples consisted of two sequential centrifugation steps; the tube was firstly centrifuged at 2400 rpm (708 g) for 10 min; thereafter, the plasma and “buffy coat” were collected and transferred using a micropipette to a new tube for a second centrifugation at 3600 rpm (1594 g) for 15 min. Most of the platelet poor plasma (PPP) was discarded and the platelet pellet was re-suspended in a small fraction of PPP to make 2 mL of the final volume sample. The gelation of L-PRP was initiated by adding 20 µL of 10% (*w*/*v*) calcium chloride in Millipore water per milliliter of L-PRP, and the gelled product was subsequently placed in a 6-well-plate containing 5 mL of serum-reduced media.

#### 4.3.2. P-PRP Preparation

For the P-PRP preparation, 10 mL of blood was centrifuged at 1800 rpm (398 g) for 8 min in order to fractionate the blood into three different layers. Thereafter, 2 mL of superficial platelet poor plasma (PPP) were discarded and 2 mL of platelet rich plasma were collected with a micropipette, with care taken to avoid turbulence and prevent buffy coat aspiration. Similarly to the L-PRP preparation, gelation was performed by adding 20 µL of 10% (*w*/*v*) calcium chloride in Millipore water per mL of P-PRP, and the gelled product was subsequently placed in a 6-well-plate containing 5 mL of serum-reduced media.

In the subsequent characterizations of the blood products, nine samples obtained from three different animals (*n* = 3 for each animal) were assayed.

### 4.4. Hemoderivative Product Characterization

#### 4.4.1. Thrombocyte Quantification

The platelet counts of the whole blood and hemoderivative preparations were determined by using an automated haematology analyser (Cell Dyn 3500, Abbot. Wiesbaden, Germany). Briefly, the whole blood was collected in 10 mL citrated tubes and manually stirred to prevent coagulation. For the anticoagulated blood products (L-PRP and P-PRP), the volume of interest (after processing but not crosslinked) was placed in citrated tubes. For the fresh products (i.e., i-PRF/A-PRF), once the centrifugation process was completed, the fraction of interest transferred into citrated tubes to prevent coagulation. Thereafter, the tubes were placed in the analyzer in order to measure platelet number per sample by electrical impedance. The results were collected from three different samples from three different animals.

#### 4.4.2. Blood Product Conditioned Media

**Figure 9:**
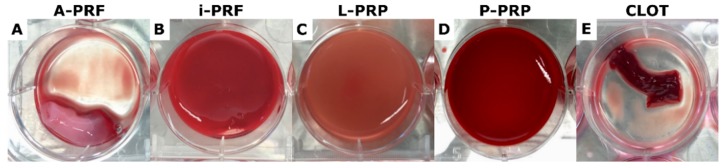
Morphology of the hemoderivative products placed in 6-well plates after three days incubation in media. Note that (**B**) (i-PRF), (**C**) (L-PRP) and (**D**) (P-PRP) coagulated in the 6-well-plate, whereas (**A**) (A-PRF) and (**E**) (CLOT) were already clotted in the blood collection tubes.

After blood collection and hemoderivative preparation (A-PRF, i-PRF, L-PRP, P-PRP and blood clot), samples were transferred to 6-well plates in 5 mL of serum-reduced media (Figure 9). The blood products were then stirred at 65 rpm on an orbital plate shaker (ZWYC-290A, Labwit Scientific Pty Ltd. Ashwood VIC, Australia) placed in the incubator at 5% CO_2_ atmosphere and 37°. After three days of incubation, the media was extracted and aliquoted at different concentrations (20%, 40%, 60%, 80% and 100% *v*/*v*) using serum-reduced α-MEM as a diluent. Thereafter, the samples were stored at −80 °C until used for the subsequent characterisation and biological assays. For measuring growth factor release from the various hemoderivatives, another set of samples were maintained for 1, 3, 7 and 14 days of incubation in 5 mL of serum-reduced α-MEM. All of the media was collected and replaced with the same volume of fresh media at each timepoint. The collected media was subsequently frozen at −80 °C until further analysis.

#### 4.4.3. Growth Factor Release (ELISA)

Enzyme-linked immunosorbent assay kits (ELISA) were purchased from Preprotech-Lonza (Mount Waverly VIC, Australia) and were used to quantify the amounts of IGF -I (900-K170), PDGF-BB (900-K04), VEGF (900-T10) and BMP-2 (900-T255) released in 5 mL serum-reduced α-MEM at 1, 3, 7 and 14 days. For each time point, three specimens from three different animals were analysed (hence, *n* = 9). Absorbance was measured at 405 nm wavelength for IGF-I and PDGF-BB. BMP-2 and VEGF were set at 450 nm in a microplate absorbance reader (POLARstar Omega. Offenburg, Germany) following the manufacturer’s protocol.

#### 4.4.4. Isolation of Human Osteoblasts

The impact of the blood product exudates (released at three days post-immersion) at various concentrations (at 20%, 40%, 60%, 80% and 100% *v*/*v*) was assessed on primary human osteoblasts. To this end, cancellous bone particles were collected from consenting patients who had undergone oral surgery as previously described [41]. Briefly, the cancellous bone fragments were washed three times with phosphate buffered saline containing 1% antibiotics (100 U/mL penicillin G, 100 μg/mL streptomycin). The bone fragments were then transferred into a T25 tissue culture flask with α-MEM containing 10% FBS and 1% antibiotics and cultured until cells migrated out of the bone fragments. Unless stated otherwise, all in vitro experiments were carried out with the following method: the osteoblasts (from Passage 2 to 4) were seeded in 24-well plates at a density of 10,000 cells/cm^2^ (hence each well received 20,000 cells) and cultured for 24 h before being incubated with the various concentrations of blood product media (500 µL/well). Thereafter the media was changed thrice a week.

#### 4.4.5. Cell Viability

Osteoblast viability was assessed at 24 and 72 h post-incubation in the blood product media. The control group consisted of cells cultured in serum-reduced α-MEM. A Live/Dead assay^®^ (Life Technologies Mulgrave VIC, Australia) was utilised to quantify cell viability as per the manufacturer’s instructions. Briefly, the cells were washed three times in PBS prior to assay. Live cells were stained with 0.02 mg/dL Calcein-AM (Thermo Fisher Scientific Inc. Newstead QLD, Australia) and dead cells were stained with 0.05 mg/dL Propidium iodine (Sigma-Aldrich. Castle Hill NSW, Australia) following incubation for 15 min at 37 °C. The fluorescence staining was visualised using confocal microscopy (Nikon Eclipse T*i*-E. Nikon Instruments INC. Melville NY, U.S.A) at maximum excitation/emission wave lengths of 493 nm for FDA and 540 nm for PI. One representative fluorescence image of each well was taken and further processed with ImageJ^®^ software (ImageJ National Institutes of Health. Bethesda MD, USA) in order to determine the percentage of live and dead cells. For each hemoderivative concentration, nine samples obtained from three different animals (*n* = 3 for each animal) were utilised.

#### 4.4.6. Metabolic Activity

Osteoblast metabolic activity was assessed at 24 and 72 h in the different blood product media. The control group consisted of cells cultured in 1% FBS α-MEM. Alamar Blue^®^ (Thermo Fisher Scientific Inc. Newstead QLD, Australia) was applied to quantify the cell metabolic activity following the manufacturer’s instructions. Briefly, cells were seeded onto 24-well plates and cultured for 24 h. Cells were washed three times in PBS, prior to being incubated with various concentrations of blood product media. After 24 and 72 h, 10 μL vol of Alamar blue^®^ was added per well and incubated for 4 h in the incubator at 5% CO_2_ atmosphere and 37 °C. The reacted Alamar solution was then removed and 100 µL aliquots in triplicate were transferred to a 96-well plate prior to absorbance reading at 570 nm in a microplate reader (POLARstar Omega, Offenburg, Germany).

#### 4.4.7. Cell Migration Assay

Osteoblasts seeded onto 24-well plates were cultured to confluence in expansion media. A scratch in the centre of each well was made with a 200 µL sterile pipette tip and washed three times with PBS to remove the detached cells and debris, as previously reported [51]. Scratch standardisation was verified at three different locations upon creation, and it was determined that the dimensional variation was below 5% (250 ± 9 µm). The cells were further incubated with the various concentrations of blood product media while the control group was cultured in serum-reduced α-MEM and cell migration was assessed at 6 and 24 h. For each well, the initial and final scratch area was calculated using Tscratch software (CSElab. ETH Zurich, Switzerland) in order to calculate the percentage of open areas.

#### 4.4.8. Mineralization Assay

Two exudate concentrations were selected (20% and 40% *v*/*v*) for assessing the mineralisation potential of the hemoderivative products at 14 and 21 days. The osteoblasts seeded onto 24-well plates were cultured to confluence in expansion media. The media was then replaced with 500 µL of the 20% and 40% hemoderivative exudates supplemented with an osteogenic factors cocktail (10 mM β-glycerophosphate, 100 mM dexamethasone and 0.2 mM ascorbic acid (Sigma-Aldrich. Castle Hill NSW, Australia), or with serum-reduced α-MEM also supplemented with the same osteogenic factor cocktail (osteogenic media), 24 h post-seeding. Basal media containing serum-reduced α-MEM and 1% antibiotics without osteogenic factors was applied as a control. The cells were further cultured for 14 and 21 days and the media was changed twice a week. Alizarin red staining was utilised to measure osteoblast mineralisation, as previously described [52]. Briefly, after 14 and 21 days, cells were fixed in 96% cold methanol for 15 min and stained with 0.2% alizarin red solution (Alizarin Red S. Sigma-Aldrich. Castle Hill NSW, Australia) in water (pH 4.2) at room temperature for 1 h. The plates were washed five times with PBS until a clear rinsing solution was obtained and images were taken using optical microscopy (Nikon Eclipse. Nikon Instruments INC. Melville, NY, USA). Alizarin red mineralised nodules were dissolved by incubating the samples in 1 mL of 10% cetylpyridinium chloride (Sigma-Aldrich. Castle Hill NSW, Australia) buffer for 1 h. The solution was then removed and 200 µL aliquots in triplicate were transferred to a 96-well plate prior to reading at 405 nm in a microplate reader (POLARstar Omega, Offenburg, Germany).

### 4.5. Statistical Analysis

Statistical analysis was conducted by one-way ANOVA, with Bonferroni post-hoc testing, using Graphpad Software vs. 7 (Graphpad Software. La Jolla, CA, USA). Statistical significance was considered at value *p* < 0.05. All data were expressed as mean ± standard deviation. Pearson correlation was performed to analyse potential correlations between: (1) cell viability at 1 or 3 days and 20–100% dilutions of the different growth factor released; and (2) Alizarin red relative values and the different BMP-2 concentrations of A-PRF, i-PRF, P-PRP and L-PRP.

## 5. Conclusions

In order to address the challenge of comparing different blood products, due to technical variations in their preparation, which account for their inconsistent performances, we utilised standardised and clinically relevant preparation protocols to enable a systematic assessment of the biological characteristics of four hemoderivatives (A-PRF, i-PRF, L-PRP, P-PRP and blood clot). Our findings demonstrate that the biochemical composition of these products is highly complex and not necessarily related to the total amount of platelets. This may be attributed to the use of either fresh or anticoagulated blood, variation in *g*-force, time, technical specifications and selective inclusion–exclusion of the buffy coat layer. This compositional complexity was further translated to heterogeneous in vitro performance, whereby high concentrations of fresh blood product appeared to be detrimental to cell viability and migration, raising questions about its current clinical application and/or whether in vitro results can be translated to in vivo performance. We also demonstrated that, at a low concentration, i-PRF (≈60 pg/mL) was the best performing product for the enhancement of mineralization, and therefore may be the candidate of choice for tissue engineering approaches utilizing autologous cues to promote bone regeneration.

## Figures and Tables

**Table 1:** Platelet recovery and growth factors levels for the different hemoderivative preparations. Data are expressed as mean ± standard error. ( ^1^ ) Indicates first and ( ^2^ ) second centrifugation step. Stars (*) represent significance difference compared to total blood (*p* < 0.0001).

Blood Product	*g*	Processing Time (min)	Platelet Recovery	Growth Factor Release 3 Days (pg/mL)			
TOTAL BLOOD	-	-	362 ± 22 × 10^3^	**IGF-I**	**PDGF-BB**	**VEGF**	**BMP-2**
A-PRF	208	8	307 ± 14 × 10^3^	265.2 ± 22.2	109.9 ± 3.4	90.3 ± 4.0	664.2 ± 37.6
i-PRF	60	3	336 ± 18 × 10^3^	66.6 ± 8.4	110.6 ± 2.5	142 ± 4.0	308.2 ± 15.9
L-PRP	708 ^1^/1594 ^2^	10 ^1^/15 ^2^	1.82 ± 17 × 10^6^ (*)	823.6 ± 24.6	261.4 ± 11.3	113.5 ± 5.1	638.2 ± 69.8
P-PRP	398	8	934 ± 32 × 10^3^ (*)	165.9 ± 2.9	109.1 ± 4.2	124.7 ± 5	652.9 ± 52.8
CLOT	-	5	326 ± 32 × 10^3^	9.8 ± 1.1	9 ± 1.1	68.4 ± 9.2	645.6 ± 41.4

## Abbreviations

A-PRF	Advance Platelet rich Fibrin
i-PRF	Injectable Platelet Rich Fibrin
P-PRP	Pure Platelet Rich Plasma
L-PRP	Leucocyte Platelet Rich Fibrin
CLOT	Thrombus, colloquially called a blood clot
IGF-I	Insulin-like growth factor 1
VEGF	Vascular endothelial growth factor
PDGF-BB	Platelet-derived growth factor subunits BB
BMP-2	*Bone morphogenetic protein 2*
